# Establishing marine protected areas in Sweden: Internal resistance versus global influence

**DOI:** 10.1007/s13280-017-0932-8

**Published:** 2017-07-29

**Authors:** Kjell Grip, Sven Blomqvist

**Affiliations:** 1 Department of Ecology, Environment and Plant Sciences, Stockholm University, 106 91 Stockholm, Sweden; 2 Mandelblomsgatan 11, 745 36 Enköping, Sweden

**Keywords:** Conservation, Marine nature reserve, Protected area, Sustainable use

## Abstract

In the past decade, marine protected areas (MPAs) have become an increasingly used tool for science-based conservation and adaptive management of marine biodiversity and related natural resources. In this review paper, we report on rather complete time-course series (55 years uninterrupted) focusing on comparison of the strong difference, in number and area, in establishing marine (56 MNRs) and terrestrial (4284 TNRs) nature reserves in Sweden versus marine (7001 MPAs) and terrestrial (132742 TPAs) protected areas globally. Sweden appears to follow the overall global time trends. The large backlog of MPAs in relation to TPAs is due to several possible reasons, such as (i) unclear marine jurisdiction, (ii) marine conservation policies and programs developed later than terrestrial, (iii) higher costs for marine conservation management, (iv) conflicts in marine conservation, especially the fishery, and (v) the general public's historically weak awareness of the status of the marine environment.

**Electronic supplementary material:**

The online version of this article (doi:10.1007/s13280-017-0932-8) contains supplementary material, which is available to authorized users.

## Introduction

World nature conservation has a long history. In the United States, the conservation movement to preserve and protect America’s wildlife, wild land, and other natural resources developed gradually between 1850 and 1920 (van Hise 1910; Gubay 1995). In Sweden, a similar development started in the late 1800s and early 1900s, and with the first national parks established in 1909 (Högdahl 1910). However, it was really only after the Second World War that modern Swedish nature conservation was established (Wramner and Nygård 2010).

### Modern history in brief

In the 1960s, the national Swedish nature conservation organization was built up. A solid jurisdictional and administrative foundation was laid by the 1964 Report of the Government Committee on Natural Resources (Ministry of Agriculture 1967) and the 1964 Nature Conservation Act. This Report and the Act formed the standard for the care of the natural environment, environmental protection, nature conservation, and sustainable management of natural resources in the country.

Negative effects of human activities were first recognized while exploiting terrestrial natural resources. The need for nature conservation and a wider establishment of terrestrial nature reserves (TNRs) had become obvious in Sweden in the 1960s (Ministry of Agriculture 1962), as in many other countries (Mani 1998). In contrast, the open sea was still regarded more or less as an infinite and inexhaustible resource, even though overfishing tendencies had been noted much earlier (Coll et al. 2008).

In the 1960s and 1970s, similar and synchronous developments in nature conservation legislation happened internationally, including also nature conservation of marine milieus. For instance, in New Zealand the Marine Reserve Act became law in 1971, in the USA the National Marine Sanctuaries Act was established in 1972, and in Australia the Great Barrier Reef Marine Park Act was declared in 1975. In 1985, a milestone was passed when the International Union for Conservation of Nature (IUCN) launched its first Marine Conservation Programme. For the first time, a model was established, demonstrating how conservation and development could protect marine and coastal species and ecosystems, and enhance awareness of marine and coastal conservation issues (Kelleher and Kenchington 1992). In 1992, marine nature conservation and biodiversity was introduced in the new HELCOM and OSPAR conventions. As late as in 1997, the first International Symposium on Marine Conservation Biology was held.

**Fig. 1:**
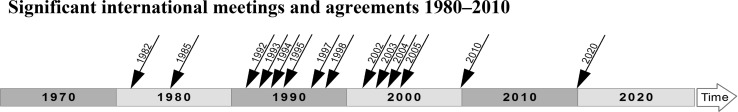
Some influential international meetings with agreements and targets that globally and regionally have contributed to increase the designation and establishment of MPAs. *1982* UNCLOS adopted; *1985* First Marine Conservation Programme of IUCN; *1992* UNCED conference in Rio de Janeiro, Chapter 17 of Agenda 21, and marine nature conservation introduced in the new 1992 HELCOM and OSPAR Conventions; *1993* UN agencies formed the Sub-committee on Oceans and Coastal Areas of the Administrative Committee on Coordination (ACC SOCA); *1994* UNCLOS entered into force; *1995* The Jakarta mandate and the Swedish membership of the EU; *1997* The First International Symposium on Marine Conservation Biology of the Society for Conservation Biology; *1998* COP/CBD adopted the first work program on marine and coastal biodiversity; *2002* The World Summit in Johannesburg agreed to “establish by 2004 a regular process under the United Nations for global reporting and assessment of the state of the marine environment, including socioeconomic aspects”; *2003* UN Oceans. United Nations High-Level Committee on Programmes approved the creation of an Oceans and Coastal Areas Network; *2004* The Regular Process established; *2005* In the mid-2000s, the EU Natura 2000 in the marine environment began to be applied (No formal decision); *2010* The CBD target of 10% ocean protection by 2020 was set in Nagoya, Japan; *2020* The 10% ocean protection target to be reached

In and around the 1990s, the issue of marine conservation and the need for marine protected areas (MPAs) were raised at a series of regional and global conferences (Table 1; Fig. 1), which lead to several significant conventions and agreements. It was also noted in increased number of scientific publications devoted to MPAs (Jones 2002).

**Table 1:** Abbreviations

CAB	County Administrative Board
CBD	Convention on Biological Diversity
EU	European Union
EEZ	Exclusive economic zone
HELCOM	Helsinki Commission
ICAM	Integrated Coastal Area Management
IUCN	International Union for Conservation of Nature
MNR	Marine Nature Reserve
MPA	Marine Protected Area
MSP	Marine Spatial Planning
NGO	Non-Governmental Organization
NPP	National Physical Planning
OSPAR	OSPAR Commission
SCB	Statistic Sweden
SEPA	Swedish Environmental Protection Agency
SNV	National Environmental Protection Board, Sweden (pre-1990)
SwAM	Swedish Agency for Water Management
TNR	Terrestrial Nature Reserve
TPA	Terrestrial Protected Area
UN	United Nations
UNCED	United Nations Conference on Environment and Development
UNCLOS	United Nations Convention on the Law of the Sea
UNEP–WCMC	United Nations Environment Programme–World Conservation Monitoring
WDPA	World Database on Protected Areas

Particularly important for the global and national nature conservation development has been the influence of the 1992 United Nations Conference on Environment and Development (UNCED), in Rio de Janeiro, including Agenda 21, the establishment of the Convention on Biodiversity (CBD), and marine-related work/agreements under the convention. Chapter 17 of Agenda 21 is focused on the oceans. The Jakarta Mandate on coastal and marine biodiversity (CBD 2000) is the global consensus on the significance of marine and coastal biodiversity. The key elements in its work program are protected marine and coastal areas and Integrated Coastal Area Management (ICAM), as related to marine spatial planning.

In Nagoya 2010, the CBD adopted the Strategic Plan for Biodiversity 2011–2020 and its Achi Biodiversity targets. Number 11 of these targets enhances that 10% of coastal and marine areas (Spalding et al. 2010) and 17% of terrestrial areas and inland waters, especially areas of particular importance for biodiversity and ecosystem services, should be protected by 2020. Related to this target and Agenda 21 is the United Nations (UN) 2030 Agenda for Sustainable development with Target 14 to conserve and sustainably use the oceans and seas.

### Aim

In the present review paper, we evaluate and analyze the time-course of designation and establishment of marine nature reserves (MNRs) (Appendix S1; Table S1) and TNRs in Sweden, from the 1960s up to present. We analyze the constraints and conflicts, concerning the use and management of marine resources (Redpath et al. 2015a) associated with the establishment of MPAs. The aim of our study is to explain the slow establishment of MNRs, why they are so few compared with the TNRs, and why it was not until the 1990s and 2000s that MNRs were recognized and promoted as a vital marine conservation tool. We also describe and compare the time-course of MNRs’ and TNRs’ establishment in Sweden with the corresponding slow establishment of MPAs and Terrestrial Protected Areas (TPAs) globally reported to the WDPA (Carr et al. 2003; Toropova et al. 2010).

## Materials and methods

In the 1980s, conservation biology became a scientific discipline of its own (Meine et al. 2006). As a research field, it was first developed in the terrestrial environment and was only much later applied to marine conservation and natural resource management.^1^ Marine spatial planning (MSP) and ecosystem-based management have attracted much increased research interest, particularly after the 1992 UNCED (Crowder and Norse 2008). This was a response to the need to understand the ecological, social, and economic linkages between conservation and ocean governance,^2^ and to find practical solutions to sustainable use of marine resources and to save the marine biodiversity (Norse and Crowder 2005; Dickman 2010; Redpath et al. 2015b). With regard to governance, there are different governance types of the protected areas: governance by government, shared governance, private governance, and governance by indigenous people and local communities (Borrini-Feyerabendance et al. 2013).

### Marine protected areas defined

The International Union for Conservation of Nature (IUCN) definition of a protected area is compatible with the CBD definition of a protected area: *A protected area is a clearly defined geographical space, recognized, dedicated and managed, through legal or other effective means, to achieve the long*-*term conservation of nature with associated ecosystem services and cultural values.*

According to the IUCN, the definitions of protected areas apply to both marine and terrestrial areas and are classified in different categories (I–VI) according to their management objectives, for instance, strict or multiple-use protection (Appendix S1). MNRs and TNRs fall mainly within one of the six management categories of protected areas (category Ia), as defined by the IUCN (Dudley 2008) and reported to the joint IUCN and United Nations Environment Programme (UNEP)–World Conservation Monitoring Center (UNEP–WCMC), and the World Database on Protected Areas (WDPA) (Appendix S1). These categories are recognized by international bodies such as the United Nations (UN) and by many national governments as the global standard for defining and recording protected areas and as such are increasingly being incorporated into government legislation.

### Regional sea areas studied

**Fig. 2:**
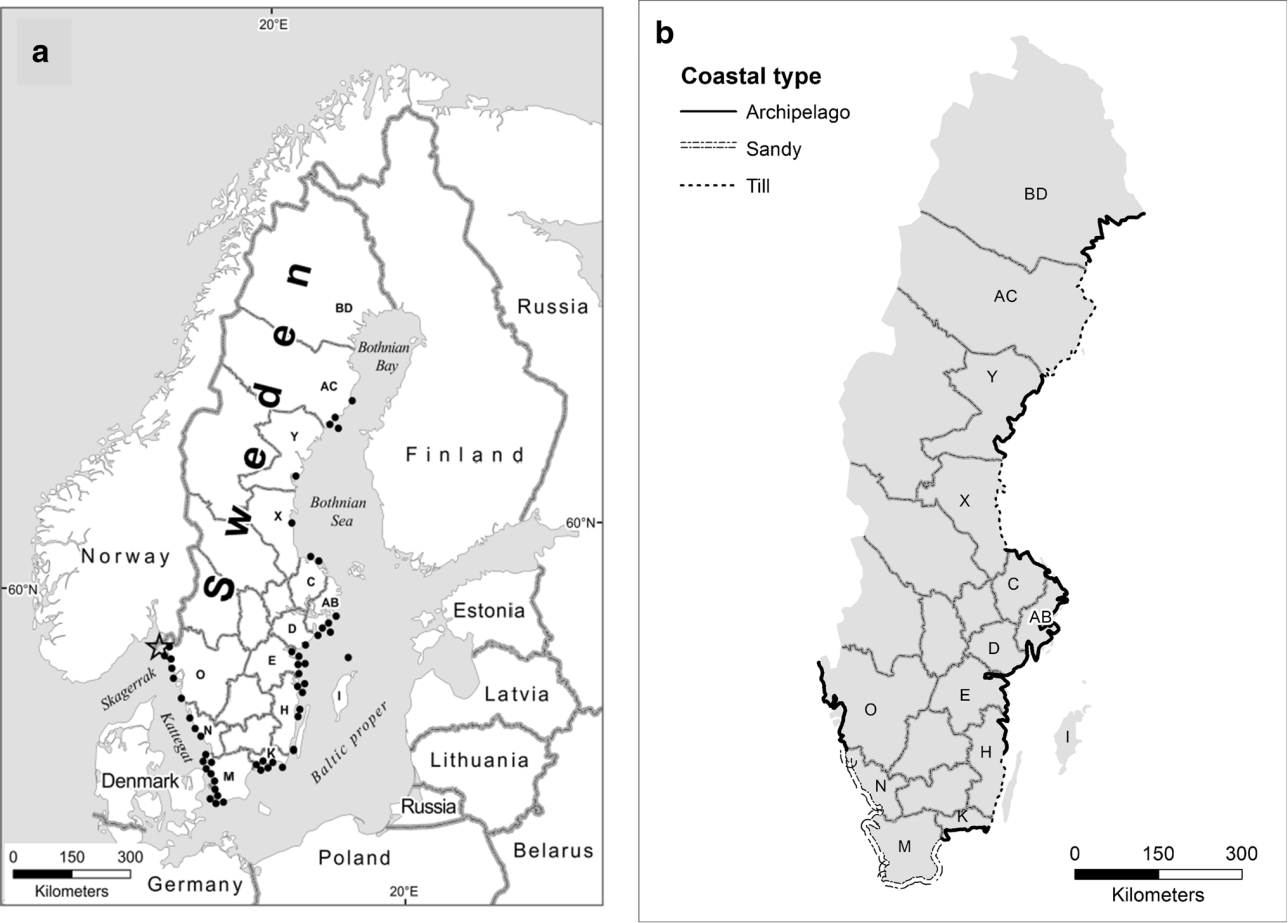
**a** Sea areas surrounding Sweden. Marine nature reserves (*black dots*) distributed along the Swedish coast in 2014. *Star* indicates Sweden’s single Marine National Park (Kosterhavet). In 2015, a further four MNRs were registered (see Appendix S1; Table S2). The acronyms of coastal counties are marked in capitals and explained in Appendix S1. **b** Geographical overview of the Swedish coastal types. *Source* Ministry of Physical Planning and Local Government, 1971

The sea areas surrounding Sweden are the semi-enclosed brackish Baltic Sea in the east and the transitional sea areas of the Kattegat and Skagerrak in the west (Fig. 2a). About 82 million people in 14 countries inhabit the drainage basin of the Baltic Sea when the Kattegat is excluded (Sweitzer et al. 1996). Around the Kattegat–Skagerrak, another 6 million people live in three countries (Persson and Kullander 2011). Much of the coastal area is industrialized or used for agriculture and forestry.

The marine biodiversity is high in the Skagerrak and decreases toward the south and into the Baltic to a minimum in the northern Gulf of Bothnia, where only few marine species are found (Ojaveer et al. 2010; Zettler et al. 2014). Due to differences in salinity, temperature, and bottom conditions, resident organisms form different biotopes and habitats, some of which are of certain conservation value, for example: eelgrass meadows, kelp forests, brown macro algae belts, cold water corals, and breeding and nursery areas (Gray 1997; HaV 2016).

### Marine jurisdiction

The international legal marine framework on rights and responsibilities in the sea is the United Nations Convention on Law of the Sea (UNCLOS) (Appendix S2; Fig. S2).

#### The Swedish nature conservation legislation and marine protected areas

In Sweden,^3^ the responsibility to protect marine and terrestrial areas is decentralized to the regional County Administrative Boards (CABs). In Sweden, 14 CABs (out of a total of 21) manage a marine coast (Fig. 2a, b). At the central level, the Swedish Environmental Protection Agency (SEPA) (formerly the National Environmental Protection Board, SNV) and the Swedish Agency for Water Management (SwAM) have coordinating roles. Municipalities can establish their own nature reserves. Out of a total of 290 municipalities in Sweden, 87 are located at the sea coast.

According to the Swedish nature conservation legislation, the Environmental Code (Ministry of Environment 2001), there is no legal difference between establishing marine and terrestrial nature reserves. The restrictions on water and land use required to achieve the purpose of the reserve can be decided. Hence, a MNR can be established as a strict reserve, or be open for multiple use. MNRs can be established from the shoreline out to the territorial boundary. MNRs cannot be established in the Exclusive Economic Zone (EEZ), but the government can establish a marine protected area in the EEZ, according to the Law of the Swedish Economic Zone. So far, no protected area is established according to this law.

The classification of marine nature reserves is built on the list of “Marina nature reserves” by SwAM and reported to the Statistic Sweden (SCB) (Appendix S1; Table S1). These MNRs may include land areas, but the basic part is focused on the water part of the MNR and has a marine purpose.

There are other categories of marine protected areas according to the Environmental Code, for example, national parks, protected areas for seals and birds, and biotope protected areas. Their management objectives are different. There are also specially protected areas, such as designated EU Natura 2000 sites according to the Birds and Habitats directives, Ramsar sites according to the Ramsar Convention, and HELCOM and OSPAR MPAs according to the Helsinki and OSPAR conventions (Appendix S1; Fact Box).

### Data accessibility

Sweden’s well-developed public data records and the legal *Principle of Public Access* (Ministry of Justice 1949) to archived records have been a precondition for the present study. This principle offers access to a most detailed documentation on designation and establishment of MNRs and TNRs, filed and archived by national and regional Swedish authorities. These files include opinions and comments expressed in connection with the establishment of a proposed nature reserve by the different authorities involved, interested organizations, property owners, and the general public. The character and extent of information obtained in the files differ between CABs, why quantification of opinions on various issues sometimes might deviate.

### Data collection

Our compiled data are based on annual unit records of MNRs and TNRs in Sweden, as well as MPAs and TPAs globally. The studied time period (55 years uninterrupted) extends from 1960, up to and including 2015 for MNRs and TNRs in Sweden, and to 2013 for MPAs and TPAs globally. Information on the number and area of MNRs and TNRs up to 1998 was obtained from the SEPA, and thereafter from the Statistics Sweden. In May and June 2012, all 14 coastal CAB officials in Sweden were visited and interviewed for the present study. Using a set of pre-prepared questions, the CAB officials were interviewed focusing on their experiences of establishing MNRs and TNRs. The files of all MNRs and the single marine national park established, up to and including May 2012, were recorded with respect to the decided regulation and comments from concerned authorities, interested stakeholders, and the public, including appealed decisions. In 2012, interviews on the experiences of establishing MNRs and other categories of protected areas were also made with responsible officials at the central agencies SEPA and SwAM.

Data on the global marine and terrestrial protected areas up to and including 2013 were obtained from the WDPA (IUCN and UNEP–WCMC 2013) and include all management categories of MPAs and TPAs. The WDPA underwent a major update in 2014, and the new data are therefore not compatible with former compilations (Juffe-Bignoli et al. 2014). Information on international conditions was also obtained from various databases accessible at special libraries, and through the Internet, including the Web of Science (ISI, Philadelphia), the World Bank Indicator–Biodiversity and Protected Areas, and the databases of HELCOM (Helsinki, Finland) and OSPAR (London, United Kingdom). In 2013, the Leigh Marine Laboratory in New Zealand was visited to discuss its experiences of designation and establishment of MPAs.

## Results

In Sweden, nature reserves are the most common form of nature conservation, with about 85% of the total protected area in the country (SEPA 2010, 2015). From the start of modern nature conservation in the 1960s, the main focus of the CABs was on the protection of terrestrial environments. The first MNR was established only in 1980.

### Distribution of MNRs along the Swedish coast

As shown in Fig. 2a, Swedish MNRs are still few, mostly small, and located close to the coast in internal waters and in the Territorial Sea. They often include land areas, and private and public waters. According to the studied documentation, the majority of the early MNRs was established as a water body associated with a pre-existing or simultaneously designated protected terrestrial archipelago area. Remarkably, the natural conservation values below the sea surface in these areas were seldom addressed or investigated by the CABs before the decision was taken. Guidelines for marine nature inventories had not been developed by SEPA at that time, unlike guidelines for terrestrial nature inventories (SNV 1975). Conservation conflicts were commonly reported, especially with property owners and the fishery. Today, guidelines for fishing in marine protected areas have been developed by SwAM (HaV 2013).

Morphologically, the Swedish coast comprises archipelago coasts of mostly firm bedrock, coasts rich in till and lag deposits, and open sandy coasts (Fig. 2b).

#### Low rate of establishing MNRs in Sweden

**Fig. 3:**
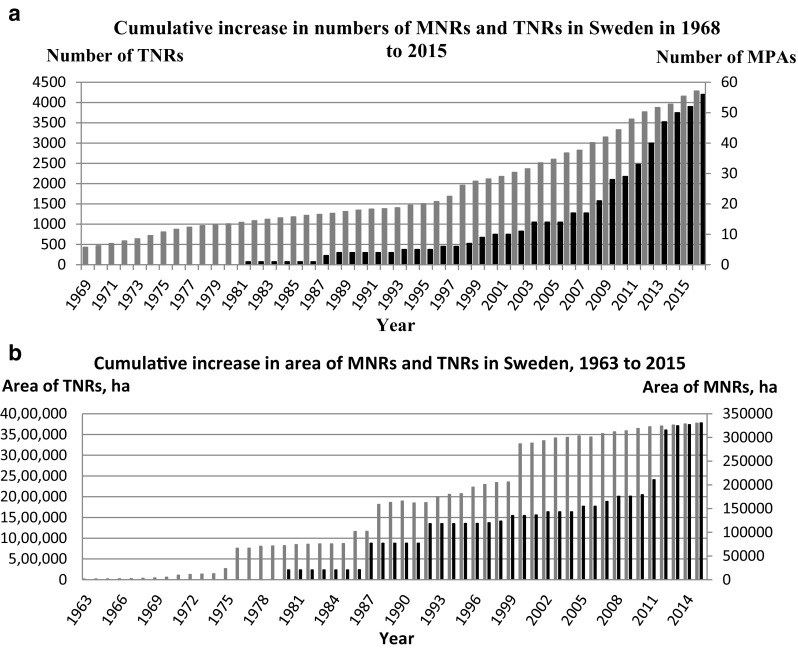
**a** Time-course of cumulative increase in the number of established terrestrial (*gray*) and marine (*black*) nature reserves in Sweden 1968–2015. Note the different scales of y-axes. *Source* Statistics Sweden and the SNV Yearbook, 1967–1979). **b** Time-course of cumulative increase in the area of terrestrial (*gray*) and marine (*black*) nature reserves in Sweden, 1963–2015. Note the different scales of y-axes. *Source* Statistics Sweden and the SNV Yearbook, 1967–1979

By 2015, Sweden had 56 MNRs (Fig. 3a; Appendix S1; Table S1), covering about 4.1% of the Swedish marine sea territory and 4.6% if the one and only Marine National Park (Kosterhavet) is included. In contrast, there were 4284 TNRs covering about 9.3% of the Swedish land territory (Fig. 3a) and 28 terrestrial national parks covering about 1.5% of the Swedish land territory (SCB 2015). In 1990, the number of MNRs amounted to 0.2% of all the TNRs, while in 2000 the corresponding value was 0.4% and in 2015 it was 1.3%. Over time, the ratio between the numbers of MNRs and TNRs has thus increased only slightly, as shown in Fig. 3a.

There is a small increase in the number of MNRs up to around 2005, followed by an even larger increase. This is reflected in the Government document 2004/05:173 *A national strategy for the marine environment* and budget allocation for marine nature conservation (Fig. 3a). For the TNRs, there is a steady increase from the 1960s up to 1998 when a faster increase started much as a response to the 1995 Action Plan for biodiversity (SEPA 1995), and proper funding by the government.

#### Areal increase of MNRs in Sweden

The cumulative areal increase of Swedish MNRs from 1963 to the present followed a stepwise pattern of increase (Fig. 3b). With the exception of 1987 and 1992, when two large MNRs (Salvorev–Kopparstenarna, I county and Falsterbohalvöns havsområde, M county) were established (Appendix S1; Table S1), there were only minor increases up to 2007. Then, a more rapid increase followed, especially after 2010, reflecting a trend of establishing larger Swedish MNRs (Fig. 3b). Of the seven largest MNRs (>10 000 ha), four were established in 1980–2008, and the other three plus the Marine National Park, after 2008 (see Appendix S1; Table S1). In 2015, the three largest MNRs established in 2009 and later accounted for 43% of the area of all MNRs. In 1990, the total area of MNRs was found to amount to 4.1% of the area of all the TNRs, 4.1% in 2000, and 8.7% in 2015. The size distribution of MNRs is shown in Table 2.

**Table 2:** Frequency distribution of MNRs in different size classes in Sweden, in 2015

Area of MNR (ha)	Number of MNRs
<100	3
100–500	12
500–1000	7
1000–5000	23
5000–10 000	4
10 000–30 000	3
30 000–50 000	2
>50 000	2
Total 330 735 ha	56

By the end of the 1960s, the overall area of Swedish TNRs was low, but from then on there was a strong growth, with particular notable increases in 1976, 1988, and 2000 (Fig. 3b). In 1976, the extensive river system Vindelälvens naturreservat was established, and in 1988–1990 large tracts of virgin forests were protected as TNRs. In 2000, large tracts of subalpine crown forests were likewise protected (Wramner and Nygård 2010).

### Marine conservation addressed—but weak results

**Fig. 4:**
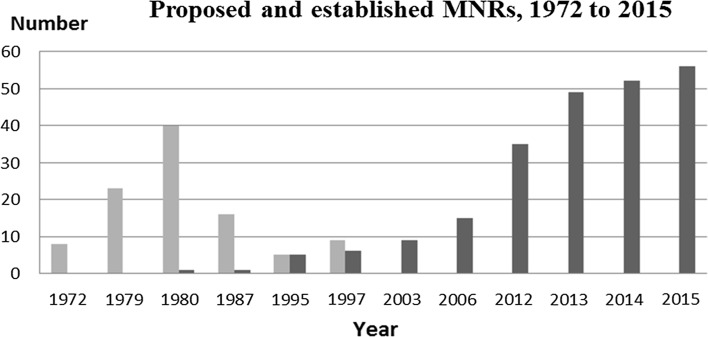
Time-course of officially proposed (*gray*) and established MNRs (*black*) in Sweden 1972–2015. *Source* Drawing based on Ministry of Physical Planning and Local Government 1971 and 1979; SEPA 1980, 1995, and 1997; Statistics Sweden and the SNV Yearbook, 1967–1979

Our review shows that the need for marine nature reserves in Sweden was raised several times from 1960 to present, but the rate of establishment of MNRs remained low compared with that of terrestrial protected areas (Figs. 3a, 4). For example, in the National Physical Planning reports of 1971 (NPP 1971) and 1979 (NPP 1979), the government presented eight and 23 marine areas worth protecting (Carlberg and Grip 1982). Yet, no MNR was established in the 1970s, compared with 423 TNRs, as shown in Fig. 3a.

In 1980, the SNV (today SEPA) presented a nationwide study on coastal and other marine areas worth protecting in Sweden (SNV 1980). About 100 highly ranked areas, including both marine and land parts, were identified. Forty of these were prioritized for protection, but without result. In this decade, Non-Governmental Organizations (NGOs) raised the issue of marine nature conservation at conferences and reports to the government and international commissions such as HELCOM. In 1987, SNV again prioritized 16 of the earlier 40 areas, in response to demands by NGOs, but still the resistance continued and very little was achieved. In the 1980s, only four new MNRs were established, as compared to 326 TNRs (Fig. 3a).

In 1993, as a follow-up to the 1992 convention of biological diversity, the Swedish Government presented a Strategy on Biological Diversity (Government Bill 1993/94:30), followed by an assignment to SEPA to develop an action plan on biological diversity. Based on this assignment, SEPA in 1995, in its Action Plan for Biological Diversity (SEPA 1995), proposed five new MNRs to be established by 1998. Linked to the action plan, criteria for the selection of MPAs were presented (Hill et al. 1997; Nilsson 1997). Also in 1993, SEPA started a parallel project, Marine reserves in Sweden (SEPA 1997) to accelerate the protection of the earlier 16 prioritized marine areas (see above). In 1997, this project resulted in a new priority list of nine MNRs of the earlier 16 prioritized marine areas to be legally protected by 1999. Again, MNRs were given low priority and the target was not reached, since in the 1990s only six MNRs were established, as compared to 807 TNRs (Fig. 3b).

In 1999, Sweden introduced a set of Environmental Objectives. Objective No. 10 includes targets for marine nature conservation and has, according to the most interviewed CAB officers, put special pressure on the CABs to act on the establishment of MNRs. A result was increased allocation of money to the CABs for establishment of MNRs (see section “Low rate of establishing MNRs in Sweden”). Still, only 23 new MNRs were established in the 2000s, as compared to 1413 TNRs (Fig. 3a). During 2011–2015, 16 MNRs were established, as compared to 516 TNRs.

### Information from the interviews with the CAB officers (in 2012)

The CAB officers’ experiences of designating and establishing MNRs can be summarized as follows:

- with regard to nature conservation, TPAs have been prioritized, especially the protection of forests;
- the CABs have small personal and financial resources for inventories, management, and supervision of MNRs. This has an influence on the establishment of MNRs. The operational supervision of MNRs is very small or absent;
- marine nature inventories and management is more costly than terrestrial. This is a disadvantage, when it comes to allocation of money to terrestrial and marine nature conservation;
- sectoral approaches in marine management have an influence on marine nature conservation;
- most CABs have no strategy for designation and establishment of marine protected areas;
- fishing in MNRs is a matter of concern. In most MNRs, fishing is under some kind of regulation. Only in a few reserves fishing is strictly prohibited; and
- generally, the public has poor knowledge of marine environmental values. Many believe that TNRs are more worthy of protection than MNRs.

### Globally protected areas

The United Nations List of Protected Areas is a compilation of all designated MPAs and TPAs in the world. The vast majority of MPAs are small and located along or close to the coast. Deep-water habitats are heavily under-represented (Juffe-Bignoli et al. 2014).

There are few published assessments of governance quality and management effectiveness. A key problem is the costs for management and enforcement.

#### Global number of MPAs and TPAs

**Fig. 5:**
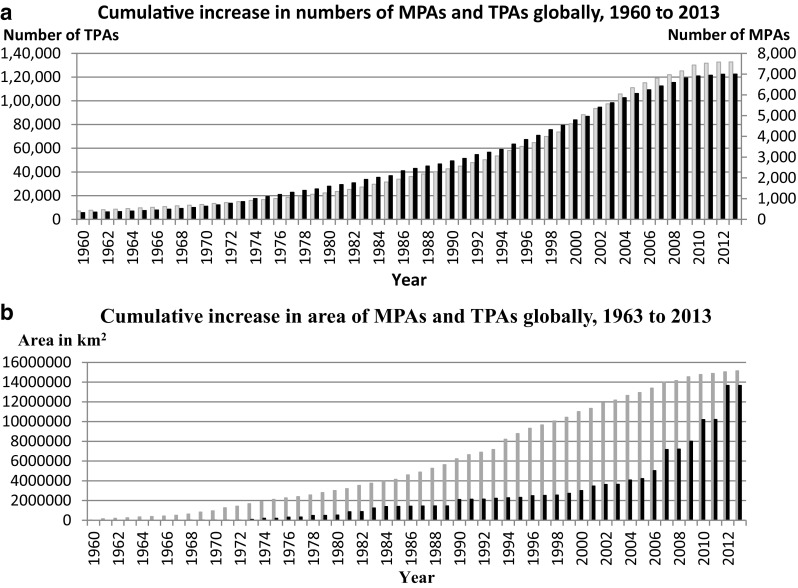
**a** Cumulative increase in the number of reported terrestrial (*gray*) and marine (*black*) protected areas at global scale, 1960–2013. Note the different scales of y-axes. The WDPA has undergone a major update in 2014 and the data beyond are not compatible with earlier data (Juffe-Bignoli et al. 2014). *Source* IUCN and UNEP– WCMC 2013. The World Database on Protected Areas. Cambridge, UK). **b** Cumulative increase in the area of reported terrestrial (*gray*) and marine (*black*) protected areas 1960–2013. The WDPA has undergone a major update in 2014 and the data beyond are not compatible with earlier data (Juffe-Bignoli et al. 2014 *Source* IUCN and UNEP-WCMC 2013. The World Database on Protected Areas. Cambridge, UK

In 2013, the world had some 7000 marine protected areas of all categories (Fig. 5a). They covered about 3.8% of the global ocean surface and 8.4% of all marine areas within national jurisdiction. On the terrestrial side, the number of TPAs is much larger, about 132 742 of all categories (Fig. 5a) covering about 10.1% of the overall land area (IUCN and UNEP–WCMC 2013).

Only about 0.25% of marine areas beyond national jurisdiction are within protected areas. In 1960, the number of MPAs corresponded to 4.6% of TPAs (calculated from Fig. 5a). In 2000, the number was 6.0% and in 2013 5.3% of TPAs. Thus, according to our study, the relative proportion of the number of MPAs to TPAs has remained rather similar over time.

For Sweden’s neighboring countries, the difference in number between MPAs and TPAs (all categories I–VI) is similar, as shown in Appendix S1; Table S3.

#### Global area of MPAs and TPAs

For the global areal growth of MPAs (Fig. 5b), the increase is slow and somewhat variable from a very low level in the 1960s, a small continuous increase up to the beginning of the 2000s. Thereafter, a more rapid increase follows, especially after 2006, when increasing numbers of larger and some very large MPAs (larger than the Swedish territory) were established (see Table 3; Wood et al. 2008). The result is that the total area of MPAs comes very close to the total area of all TPAs (Fig. 5b). In 1960, the area of MPAs corresponded to about 21% of TPAs (calculated from Fig. 5b), in 2000 27%, rising in 2013 to 90% of TPAs, or 47% of all protected areas (MPAs + TPAs). The main driving force for this trend is political, and due to commitments to international conservation targets, especially the 2010 CBD target of 10% ocean protection by 2020, but also intense domestic and international lobbying (Leenhardt et al. 2013). The percentage of MPAs and TPAs by CBD region in 2014 is shown in Appendix S1; Fig. S1.

**Table 3:** The six largest MPAs globally in 2012. *Source* Wood et al. (2008)

Country	Name	Established year	Water area (km^2^)
Australia	Great Barrier Reef	1975/1979	345 400
Ecuador	Galapagos	1996	133 000
Australia	Macquarie, Tasmana	1999	162 000
USA	Papahanaumokuakea, Hawaii	2006	360 000
Kiribati	Phoenix Island (PIPA), Pacific Ocean	2008	408 250
United Kingdom	Chagos MPA, Indian Ocean	2010	545 000
Total area			1953 650^a^

^a^ After 2012, the Cook Islands, associated with New Zealand, has created a 1 million km^2^ marine park encompassing roughly half of the country’s Exclusive Economic Zone, and the French territory of New Caledonia with a 1.4 million km^2^ multiple-use MPA. In October 2016, the Commission for the Conservation of Marine Living Resources (CCAMLR) reached consensus on a New Zealand/United States proposal to establish a large-scale marine protected area in the Ross Sea region of Antarctica. The Agreement will enter into force on December 1, 2017. The Ross Sea region MPA covers 1.55 million km^2^, of which 1.12 million km^2^, or 72%, is fully protected (no fishing is permitted). It is the world’s largest MPA (Hallett 2016)

Our review shows that the relation between the area of MNRs and TNRs in Sweden (Fig. 3b) differs strongly from the global relation between the areas of MPAs and TPAs (Fig. 5b). Still, there is a trend of establishing larger MNR areas also in Sweden. In 2015, the three largest Swedish MNRs, established after 2009, accounted for 43% of the area of all MNRs (Table 2). In 2013, the world’s six largest MPAs accounted for about 14% of the total MPA area (Table 3).

For TPAs, there was found a continuous, slow global increase from the 1970s up to mid-1980s, followed by a more rapid increase (Fig. 5b).

## Discussion

There is a slow establishment of different categories of MPAs compared with TPAs. Globally, our understanding is that the protection and management of marine ecosystems and biodiversity has developed later than terrestrial conservation policies and programs (HELCOM 2010). Still, marine conservation policies and programs are poorly developed compared to terrestrial (see section “Distribution of MNRs along the Swedish coast” and “Globally protected areas”), and generally, marine conservation continues to lag far behind terrestrial conservation.

### Why are there fewer marine than terrestrial protected areas?

Below, we discuss several constraints and conflicts associated with the use and management of marine resources and marine nature conservation which, singly or in combination, has contributed to the low establishment rate and late increase in marine protected areas (Figs. 3a, 5a; Kearney et al. 2013). We suggest five main reasons that largely explain this difference:

#### Unclear marine jurisdiction before UNCLOS

A desire to protect and conserve the marine environment is reflected in the national legislation of most countries, albeit frequently in an incomplete and inadequate way. An important reason for the low number and late start of establishments of MPAs is likely that it was only after 1994, when UNCLOS entered into force (Appendix S2; Fig. S2) that the rights and responsibilities of the coastal states became legally clear (Kelleher and Kenchington 1992; Jacobsson 2009). Also, in most countries, translating international law into national legislation takes time to implement and enforce. In Sweden, our study shows that the administrative boundaries, and thereby the management responsibilities in the sea of the municipalities and the CABs, were not determined until the 1990s, and the coastal CABs initially lacked the management competence in marine nature conservation.

Still, the rate of establishment of MPAs is low in many countries and established MPAs are often not properly managed and enforced (Barcott 2011). Enforcement and control are key problems, since the provisions of national legislative conservation acts and regulations in territorial and EEZ waters are not implemented or applied effectively to guarantee development without undermining the natural resource base (OECD 2016).

In the high seas, beyond the jurisdiction of any nation, the use of MPAs as a protection instrument has yet to be incorporated formally into international law, in order to make the establishment and implementation of MPAs in the high seas effective (Baker 2001; Houghton 2014) (Appendix S2). The Regional Seas Conventions can designate MPAs in the high seas. An example is the arrangement between the North East Atlantic Fishery Commission (NEAFC) and OSPAR regarding the collective management of high seas protected areas in the North East Atlantic (Grip 2017). However, these areas are not legally protected.

#### Late tools for integrated marine planning and management

Marine^4^ and maritime^5^ management is by tradition characterized by sectoral management (Douvere 2008; Crowder and Norse 2008; Wanfei and Jones 2013). As reported by the Swedish CAB officers interviewed, sectoral approaches often hamper the establishment of MPAs and sustainable use of resources, as sectoral management often ignores the activities of other sectors. The 1992 UNCED added a new governance and management approach for ocean and coastal decision-making to that of UNCLOS (1982) (VanderZwaag 1996). UNCED (Appendix S2) recommended and influenced coastal states to establish principles and policies for a comprehensive marine governance to ensure the sustainable use of the sea and a cross-sectoral coordinated and integrated planning and management of marine and maritime activities. After the 1992 UNCED, instruments such as Integrated Marine and Coastal Area Management, Marine Spatial Planning, and Ecosystem-Based Management slowly began to be developed by regional marine commissions and individual countries.

Today, these instruments are rather commonly used tools in marine and maritime management, including marine nature conservation, to guide decision-making by the sectors involved in addition to sector-specific laws (Grip 1992; Naturvårdsverket 2000; Carr et al. 2003; Farmer et al. 2012). This development in the planning and management of oceans and seas is reflected in the UN five-year Action Agenda 2012–2016 (UNEP 2012) and the European Union’s the Blue Paper on a European Maritime Policy (EU/EC 2007) with the Framework Directive for Maritime Spatial Planning (Directive 2014/89/EU).

#### Conflicts among resource users

Most of the interviewed Swedish CAB officers emphasized that, compared to other marine activities, the commercial fishery in Sweden has a disproportionately profound influence on the establishment of marine nature reserves. This has been the case even though most of the Swedish fishing revenue comes from fishing outside the territorial waters, whereas all MNRs are located inside. Proposals for MNRs with strict protection have usually met strong opposition from property owners (which in archipelago areas can be many), fishermen, and local municipalities (Redpath 2015b). Such conflicts have delayed or prevented the establishment of most MNRs in Sweden. Also, this is in accordance with experiences from almost all areas of the world, with conflicts and lack of cooperation between management agencies for the environment and for fisheries. One reason to this situation is the sectoral approaches. Usually, the management of fisheries and nature conservation is carried out by different agencies, under separate ministries, with different legislation and supported by different NGOs. Often, these conflicts concern whether a MNR/MPA should be strictly protected or open to traditional fishing, albeit perhaps with some restrictions (Kelleher 1999; Grip 2002; Gaines et al. 2010; Kearney et al. 2012; Redpath et al. 2015b).

MPAs are often proposed in areas where conflicting interests are already established, and encroachments on these interests are then usually not accepted (Chuenpagdee et al. 2013). Resistance to the establishment of MPAs and associated conflicts (see section “Distribution of MNRs along the Swedish coast”) are generally due to demands for restrictions of property owners’ use of the area and associated with interests, such as wind power establishment, sand and gravel extraction, fishery, aquaculture, tourism, and shipping. Basically, these conflicts in conservation are between socioeconomic factors and environmental issues (Dickman 2010; White and Ward 2010), in the sense that political processes decide on which interest should be prioritized**—**conservation or resource use. It has sometimes been claimed that MPA effectiveness is low and that MPAs do not work as a protection instrument (Benneta and Deardenc 2014), but recent research (Fogarty and Murawski 2004: Edgar et al. 2014; Halpern 2014) demonstrates that MPAs can work, in line with our experiences from Sweden.

#### Higher costs for marine conservation management

At-sea management is costly and budget constraints are common. The need for ships and advanced surveying technology means that marine conservation management generally is more demanding and expensive than the corresponding terrestrial management (McCrea-Strub et al. 2011). Funds for inventories, monitoring, control, surveillance, and enforcement of MPAs in many countries are insufficient to ensure proper implementation and management of the MPAs. The main source of MPA financing in developed countries is government budget, whereas in developing countries international donors as well as user fees can constitute an important source of MPA finance (OECD 2016).

Recent advances in photographic techniques, marine modeling, scuba diving, and submersibles (Autonomous Underwater Vehicles; Kelley et al. 2016) have made extended underwater observations more efficient and less costly (Wynna et al. 2014). However, as pointed out by several Swedish CAB officers, the higher costs for marine than terrestrial conservation still matter when it comes to allocation of financial resources for marine and terrestrial management, involving inventories, enforcement, and control of MNRs/MPAs. The experience in Sweden and elsewhere (see section “Marine conservation addressed – but weak results”) shows that the establishment and management of MNRs and MPAs are dependent on political support and proper funding to be successful (McCrea-Strub et al. 2011).

#### Low publicity, public and stakeholder support

The difficulty of observing and experiencing the underwater environment has limited public support for the designation and establishment of MNRs and MPAs. Today, the development of marine underwater technology, recreational diving, marine ecotourism, the increased media interest, and increasing media coverage of marine underwater life has enhanced public awareness, interest, and support for the protection of marine values and establishment of MPAs. However, the general public’s awareness of the management problems associated with the sea and their resources is generally low (FEI 1999; Gelcich et al. 2014).

The capability of a country to deal with its environmental problems depends to a large degree on enlightened and well-informed citizens (Bennetta and Deardenc 2014). Non-governmental organizations interested in protection of the marine and coastal environments are growing in number and strength. Globally, they already represent a strong public voice and wield considerable political influence by promoting or opposing development plans and conservation policies. Our reporting CAB officers in Sweden, as well as studies elsewhere (deFontaubert et al. 1996; Voyer et al. 2012), indicate that without effective communication, strong public participation, and community and stakeholder support, the designation, establishment, and enforcement of MNRs and MPAs will often be delayed, weakened, or even prevented.

## Final remarks

Weak jurisdiction for enforcement, strong sector authorities, budget shortfalls, faulty planning, and insufficient community support have contributed to the slow establishments of MNRs and MPAs. Commitments to new international marine conventions and agreements have helped to raise interest in marine nature conservation and have influenced the rate that which MPAs have been designated and established, globally and nationally (Appendix S2; Fig. S3). In recent years, the network of MNRs and TNRs in Sweden (Fig. 3a, b), and MPAs and TPAs regionally and globally (Fig. 5a, b) has increased considerably, both in number and areal coverage, albeit from a low level. This is a result of a growing global awareness of the need to safeguard marine biodiversity and natural resources. The still low number of MPAs underlines the need for more effective governance of how MPAs are planned, established, managed, and enforced. Also, there is a political will needed to allocate necessary funds to support the establishment and management of MPAs, in order to reach the CBD target of 10% ocean protection by 2020 (CBD 2011). In this regard, the recent trend of establishing larger areas of MPAs will contribute to reach or come closer to that goal.

## Electronic supplementary material

Below is the link to the electronic supplementary material.

Supplementary material 1 (PDF 443 kb)

## Footnotes

1 Management in organizations is the function that coordinates the efforts of people to accomplish goals and objectives using available resources (e.g., legal and economic) efficiently and effectively.

2 Governance is the sharing of policy-making competencies in a system of negotiation between nested governmental institutions at several levels (international, (supra)national, regional, and local) on the one hand and governmental actors, market parties, and civil society organizations on the other in order to govern activities at sea and their consequences (Van Tatenhove 2011).

3 In most countries, the state responsibility begins at the shoreline, but Sweden is different. The Swedish territory, out to the territorial boundary, is divided into municipalities. It means that the responsibility of a coastal municipality and a CAB covers coastal waters out to the territorial boundary. Also, in Sweden a private land property can include water out to around 300 m from the shoreline. Sweden, including the EEZ, has responsibility for about 41% of the Baltic Sea marine area (DSH 1989).

4 Marine refers to studies of the oceans and seas including their flora and fauna as well as their interaction with coastal territories and with the atmosphere. One of the major concerns of marine research is the preservation of marine ecosystems.

5 Maritime refers to industries/livelihoods related to the human use of ocean and seas resources, involving shipping, fishery, tourism, design, construction, etc.

## References

[CR1] Baker M, Bett B, Billet D, Rogers A (2001). The status of natural resources on the high-seas – an environmental perspective.

[CR2] Barcott B (2011). The unfulfilled promise of the world’s marine protected areas. Yale Environment 360.

[CR3] Bennetta NJ, Deardenc P (2014). Why local people do not support conservation: Community perceptions of marine protected area livelihood impacts, governance and management in Thailand. Marine Policy.

[CR4] Borrini-Feyerabend, G., N. Dudley, T. Jaeger, B. Lassen, N. Pathak Broome, A. Philips, and T. Sandwith. 2013. Governance of protected areas: from understanding to action. Best Practice Protected Area Guidelines Series No. 20. Gland: IUCN.

[CR5] Carlberg C, Grip K (1982). Coastal policy in Sweden—uses and protection of marine resources. Ekistics.

[CR6] Carr MH, Neigel JE, Estes JA, Andelman SJ, Warner RR, Largier JL (2003). Comparing marine and terrestrial ecosystems: Implications for the design of coastal marine reserves. Ecological Applications.

[CR7] CBD (2000). Convention on biological diversity: The Jakarta Mandate—from global consensus to global action.

[CR8] CBD. 2011. Strategic plan for biodiversity 2011–2020. Target 11. COP/10/INF/12/Rev.1. Montreal: CBD Secretariat.

[CR9] Chuenpagdee R, Pascual-Fernandez JP, Szelianszky E, Alegrte JL, Fraga J, Jentoft S (2013). Marine protected areas: Rethinking their inception. Marine Policy.

[CR10] Coll M, Libralato S, Tudela S, Palomera I, Pranovi F (2008). Ecosystem overfishing in the ocean. PLoS ONE.

[CR11] Crowder LB, Norse EA (2008). Essential ecological insights for marine ecosystem-based management and marine spatial planning. Marine Policy.

[CR12] deFontaubert, C., D. Downes, and T.S. Agardy. 1996. Biodiversity in the seas: Protecting marine and coastal biodiversity and living resources. Under the convention on biological diversity. IUCN Environmental Law and Policy Paper # 32. Washington DC: Island Press

[CR13] Dickman A (2010). Complexities of conflict: the importance of considering social factors for effectively resolving human–wildlife conflicts. Animal Conservation.

[CR14] Douvere F (2008). The importance of marine spatial planning in advancing ecosystem-based sea use management. Marine Policy.

[CR15] DSH. 1989. Swedish marine resources activities in the 1990s. Proposed overall programme. The Swedish marine resources commission, 1989:2, Göteborg, Sweden.

[CR16] Dudley N (2008). Guidelines for applying protected areas management categories.

[CR17] Edgar GJ, Stuart-Smith RD, Willis TJ, Kininmonth S, Baker SC, Banks S, Barrett NS, Becerro MA (2014). Global conservation outcomes depend on marine protected areas with five key features. Nature.

[CR18] EU/EC. 2007. Communication from the Commission to the European Parliament, the Council, the European Economic and Social Committee and the Committee of the Regions. An integrated maritime policy for the European Union, COM(2007) 575 final. Brussels, Belgium.

[CR19] Farmer, A., L. Mee, O. Langmead, P. Cooper, A. Kannen, P. Kershaw, and V. Cherrier. 2012. The ecosystem approach in marine management. EU FP7 KNOWSEAS Project.

[CR20] FEI (1999). Raising environmental awareness in the Baltic Sea area. The Finnish Environment 327.

[CR21] Fogarty MJ, Murawski SA (2004). Do marine protected areas Really Work? Georges Bank experiment offers new insights on age-old questions about closing areas to fishing. Woods Hole Oceanographic Institution. Oceanus Magazine.

[CR22] Gaines SD, White C, Carr MH, Palumbi SR (2010). Designing marine reserve networks for both conservation and fisheries management. Proceedings of the National academy of Sciences of the United States of America.

[CR23] Gelcich S, Buckley P, Pinnegar JK, Chilvers J, Lorenzoni I, Terry G, Guerrero M, Castilla JC (2014). Public awareness, concerns, and priorities about anthropogenic impacts on marine environments. Proceedings of the National Academy of Sciences of the United States of America (PNAS).

[CR24] Gray J (1997). Marine biodiversity: patterns, threats and conservation needs. Biodiversity Conservation.

[CR25] Grip K (1992). Coastal and marine management in Sweden. Ocean and Coastal Management.

[CR26] Grip K (2002). Better integration of environmental and fisheries sciences for management advice. Estuarine, Coastal and Shelf Science.

[CR27] Grip K (2017). International marine environmental governance: A review. Ambio.

[CR28] Gubay S (1995). Marine protected areas—Past, present and future. Conservation Biology.

[CR64] Hallett C (2016). Nations agree to create world´s largest marine reserve in Antarctica. Science.

[CR29] Halpern BS (2014). Conservation: Making marine protected areas work. Nature.

[CR30] HaV 2013. Vägledning. Reglering av fiske i marina skyddade områden. [Guidelines. Regulation of fishing in marine protected areas]. Swedish Agency for Water Management. Report 2013:13. Göteborg, Sweden (in Swedish).

[CR31] HaV 2016. Handlingplan för marint områdesskydd. Myllrande mångfald och unika naturvärden i ett ekologiskt nätverk under ytan. [Action plan for marine protected areas. Thriving diversity and unique natural values in an ecological network under the surface]. Swedish Agency for Water Management, Göteborg, Sweden (in Swedish).

[CR32] HELCOM. 2010. Towards an ecological coherent network of well-managed marine protected areas: Implementation report on the status and ecological coherence of the HELCOM BSPA network. Helsinki Commission, Baltic Sea Environment Proceedings No. 124B, Helsinki, Finland.

[CR33] Hill, C., K. Grip, S. Evans, I. Jansson, K. Jansson, S. Johansson and P. Jonsson. 1997. Mål och åtgärder för bevarande av biologisk mångfald i svenska havsområden [Objectives and measures for conservation of biodiversity in Swedish marine areas]. Swedish Environmental Protection Agency, Report 4599, Stockholm, Sweden (in Swedish, English summary).

[CR35] Högdahl T. 1910. Naturskyddsfrågans utveckling i Sverige [Development of nature conservation in Sweden]. The Swedish Society for Nature Conservation Yearbook, *Sveriges Natu*r 1: 8–23. Stockholm, Sweden. (in Swedish).

[CR34] Houghton K (2014). Identifying new pathways for ocean governance: The role of legal principles in areas beyond national jurisdiction. Marine Policy.

[CR36] IUCN and UNEP–WCMC. 2013. The world database on protected areas (WDPA). Retrieved 10 Oct 2016, from: www.protectedplanet.net.

[CR37] Jacobsson, M. 2009. Folkrätten, havet och den enskilda människan [International law, the sea and the individual man]. Liber, Stockholm, Sweden (in Swedish).

[CR38] Jones PJ (2002). Marine protected area strategies: issues, divergences and the search for middle ground. Reviews in Fish Biology and Fisheries.

[CR39] Juffe-Bignoli D, Burgess ND, Bingham H, Belle EMS, de Lima MG, Deguignet M, Bertzky B, Milam AN (2014). Protected planet report 2014.

[CR40] Kearney R, Buxton CD, Farebrother G (2012). Australia’s no-take marine protected areas: Appropriate conservation or inappropriate management of fishing?. Marine Policy.

[CR41] Kearney R, Farebrother G, Buxton CD, Goodsell P (2013). How terrestrial management concepts have led to unrealistic expectations of marine protected areas. Marine Policy.

[CR42] Kelleher G, Kenchington R (1992). Guidelines for establishing marine protected areas. A marine conservation and development report.

[CR43] Kelleher G (1999). Guidelines for marine protected areas.

[CR44] Kelley C, Kerby T, Sarradin P-M, Sarrazin J, Lindsay DJ, Clark MR, Conselvey M, Rowden AA (2016). Submersibles and remotely operated vehicles. Biological Sampling in the Deep Sea.

[CR45] Leenhardt P, Cazalet B, Salvat B, Claudet J, Feral F (2013). The rise of large-scale marine protected areas: Conservation or geopolitics?. Ocean and Coastal Management.

[CR46] Mani M (1998). In search of pollution havens? Dirty industry in the world economy, 1960 to 1995. Journal of Environment Development.

[CR47] McCrea-Strub A, Zeller D, Sumaila UR, Nelson J, Balmford A, Pauly D (2011). Understanding the cost of establishing marine protected areas. Marine Policy.

[CR48] Meine C, Soulé M, Noss RE (2006). A mission–driven discipline: The growth of conservation biology. Conservation Biology.

[CR49] Ministry of Agriculture 1962. Naturen och samhället [The Nature and the Society. The 1960 Investigation on nature conservation]. Government Official Report, SOU 1962: 36. Stockholm, Sweden (in Swedish).

[CR50] Ministry of Agriculture 1967. Miljöforskning [Environmental research. The 1964 Government Committee on Natural Resources]. Government Official Reports, SOU 1967: 43, and SOU 1967: 44. Stockholm, Sweden (in Swedish).

[CR51] Ministry of Environment, 2001. Miljöbalken [The Swedish Environmental Code]. Letter of Ministry, Ds 2000: 61. Stockholm, Sweden (in Swedish).

[CR52] Ministry of Justice. 1949. Offentlighetsprincipen [The Principle of Public Access in the Swedish Freedom of the Press Act, December 2nd of 1766]. The Swedish Instrument of Government, SFS 1949: 105 (Chapter 2 and 14). Stockholm, Sweden (in Swedish).

[CR53] Naturvårdsverket. 2000. Miljöinriktad fysisk planering. [Environmentally related physical planning]. Boverket and Naturvårdsverket. Swedish Environmental Protection Agency, Report 5096. Stockholm, Sweden (in Swedish, English summary).

[CR54] Nilsson, P. 1997. Kriterier för val av marina skyddade områden – en analys [Criteria for the selection of marine protected areas – an analysis]. Swedish Environmental Protection Agency, Report 4750. Stockholm, Sweden (in Swedish, English summary).

[CR55] Norse EA, Crowder LB (2005). Marine conservation biology. The science of maintaining the sea’s biodiversity. Marine Conservation Biology Institute.

[CR56] NPP. 1971. National physical planning. Ministry of housing and physical planning. Government Official Report, SOU 1971: 75. Stockholm, Sweden.

[CR57] NPP. 1979. National physical planning 2. Ministry of housing and physical planning. Government Official Reports, Part 1 SOU 1979: 54 and Part 2 SOU 1979:55. Stockholm, Sweden

[CR58] OECD. 2016. *Marine protected areas economics, management and effective policy mixes. Policy highlights*. Paris: OECD Environment Directorate. Retrieved 20 Feb 2017, from: https://www.oecd.org/environment/resources/Marine-Protected-Areas-Policy-Highlights.pdf.

[CR59] Ojaveer H, Jaanus A, MacKenzie BR, Martin G, Olenin S, Radziejewska T, Telesh I, Zettler ML, Zaiko A (2010). Status of biodiversity in the Baltic Sea. PLoS ONE.

[CR60] Persson J. and M. Kullander. 2011. SWOT– analys för Kattegat och Skagerrak [SWOT– analysis for the Kattegat and the Skagerrak]. Interreg IV A. Oxford Research. Stockholm, Sweden (in Swedish).

[CR61] Redpath SM, Gutierrez RJ, Wood KA, Sidaway R, Young JC, Redpath RM, Gutierrez RJ, Wood KA, Sidaway R, Young JC (2015). Introduction to conservation conflicts. Conflicts in conservation. Navigation towards solutions.

[CR62] Redpath SM, Sutherland WJ, Redpath SM, Gutierrez RJ, Wood KA, Sidaway R, Young JC (2015). The value of ecological information in conservation conflicts. Conflicts in conservation. Navigation towards solutions.

[CR63] SCB. 2015. Skyddad natur [Protected Nature]. Statistics Sweden. Stockholm, Sweden. Retrieved 1 Oct 2016, from: http://www.statistikdatabasen.scb.se/pxweb/sv/ssd/?rxid=417ae2e8-465a-4e41-a869-5dfe03f6c2b8.

[CR65] SEPA. 1995. Aktionsplan för biologisk mångfald [Action plan on biological diversity]. Swedish Environmental Protection Agency, Report 4463. Stockholm, Sweden (in Swedish).

[CR66] SEPA. 1997. Marina reservat [Marine nature reserves]. Swedish Environmental Protection Agency, Report 4693. Stockholm, Sweden (in Swedish).

[CR67] SEPA. 2010. Naturreservat i Sverige [Nature reserves in Sweden]. The Swedish Environmental Protection Agency. Stockholm, Sweden (in Swedish).

[CR68] SEPA. 2015. Naturreservat–vanlig och stark skyddsform [Nature reservs–a common and strong protection]. The Swedish Environmental Protection Agency. Stockholm, Sweden. Retrieved 5 April 2016, from: www.naturvardsverket.se/Var-natur/Skyddad-natur/Naturreservat/.

[CR69] SNV. 1975. Översiktlig naturinventering och naturvårdsplanering. Råd och anvisningar. [Comprehensive nature inventories and conservation planning. Advice and instructions]. National Environmental Protection Board, SNV 1975:1. Stockholm, Sweden (in Swedish).

[CR70] SNV. 1980. Inventering av värdefulla områden längs Sveriges kust [Inventory of valuable areas along the Swedish coast]. National Environmental Protection Board, SNV PM 1297. Stockholm, Sweden (in Swedish).

[CR71] Spalding M, Wood L, Fitzgerald C, Gjerde K, Torepova C, Meliane I, Laffoley D, Matthews E, Spalding M (2010). The 10% target: Where do we stand?. Global Ocean Protection: Present Status and Future Possibilities.

[CR72] Sweitzer J, Langaas S, Folke C (1996). Land use and population density in the Baltic Sea drainage basin. Ambio.

[CR73] Toropova C, Kenchington R, Vierros M, Meliane I, Torepova C, Meliane I, Laffoley D, Matthews E, Spalding M (2010). Benefits and challenges of MPA strategies. Global ocean protection: Present status and future possibilities.

[CR74] UNEP. 2012. Green economy in a blue world—a synthesis report. UNEP, FAO, IMO, UNDP, IUCN, World Fish Center, GRID Arendal, Norway.

[CR75] VanderZwaag, D. 1996. Sustainable development in the maritime sector. Ocean law and policy challenges. Dalhousie Law School and Associate, Oceans Institute of Canada. Halifax, Canada, 30 pp. (Paper presented at a symposium by the Organization of Eastern Caribbean States in St. Lucia 1996).

[CR76] Van Hise CR (1910). The Conservation of natural resources in the United States.

[CR77] Van Tatenhove J. 2011. Integrated marine governance. Environmental Policy Group, Wageningen University. MAST 2011, 10(1): 87-113.

[CR78] Voyer M, Gladstone W, Goodall H (2012). Methods of social assessment in marine protected areas: Is public participation enough?. Marine Policy.

[CR79] Wanfei Q, Jones PJS (2013). The emerging policy landscape for marine spatial planning in Europe. Marine Policy.

[CR80] White PSL, Ward AI (2010). Interdisciplinary approaches for the management of existing and emerging human-wildlife conflicts. Wildlife Research.

[CR81] Wood LJ, Fish L, Laughren J, Pauly D (2008). Assessing progress towards global marine protection targets: Shortfalls in information and action. Oryx.

[CR82] Wramner, P. and O. Nygård. 2010. Från naturskydd till bevarande av biologisk mångfald [From nature protection to conservation of biodiversity]. Commission for Communications Regulation (COMREG) Studies in Environment and Development No. 2. Södertörn University. Stockholm, Sweden (in Swedish).

[CR83] Wynna RB, Huvennea VAI, Le Basa TP, Murtona BJ, Connellya DP, Betta BJ, Ruhla HA, Morrisa KJ (2014). Autonomous underwater vehicles (AUVs): Their past, present and future contributions to the advancement of marine geoscience. Marine Geology.

[CR84] Zettler ML, Karlsson A, Kontula T, Gruszka P, Laine AO, Herkül K, Schiele KS, Maximov A, Haldin J (2014). Biodiversity gradient in the Baltic Sea: a comprehensive inventory of macrozoobenthos data. Helgoländer Marine Research.

