# Supplementary material for: Establishing marine protected areas in Sweden: Internal resistance versus global influence
Source: Ambio. 2017 Jul 29;47(1):1–14. doi: 10.1007/s13280-017-0932-8 (PMC5709261; doi:10.1007/s13280-017-0932-8)
Supplement: Supplementary file 1 — Supplementary material 1 (PDF 443 kb) [file 13280_2017_932_MOESM1_ESM.pdf]

Ambio

Electronic Supplementary Material
This supplementary material has not been peer reviewed.

Title:
Establishing marine protected areas in Sweden: Internal resistance versus global influence

Authors: Kjell Grip and Sven Blomqvist

Appendix S1

Marine nature reserves in Sweden, 2015

Table S1 Swedish marine nature reserves in 2015 by county, year of decision, water and land area in hectares, and regulation of fisheries according to different Swedish laws: FL=Fisheries Legislation, MB=Miljöbalken [the Environmental Code], MNP=Marine National Park, and – is not regulated.

Land figures indicate the range of area. The County Administrative Board scheme starts from Västra Götaland (Bohuslän) and follows the coast to Västerbotten in the north. The Norrbotten County (BD) above, lacks marine nature reserve. Source: Public records at coastal CABs and Statistics Sweden.

The number and area of MNRs may vary between years since new areas are included, merged with other nature reserves or deleted as a MNR

Nr	Marine nature reserves (MNRs)	County / Province	Year of decision	Water area, ha (Land area, ha)	Regulation of fishery
1	Gullmarsfjorden	**Västra Götaland/ Bohuslän (O)**	1999 (1983)	11 730 (4 769)	FL
2	Nordre älvs mynning	Västra Götaland/ Bohuslän (O)	2005	5 510 (1 529)	FL
3	Havstensfjorden	Västra Götaland/ Bohuslän (O)	2010	1 660 (268)	FL
4	Väderöarna	Västra Götaland/ Bohuslän (O)	2011	18 090 (150)	FL
MNP	*Kosterhavet Marine National Park*	*Västra Götaland/ Bohuslän (O)*	*2009*	*37 960 (980)*	*FL*
5	Saltö	Västra Götaland/ Bohuslän (O)	2011	200 (196)	–
6	Kosteröarna	Västra Götaland/ Bohuslän (O)	2010 (1984)	550 (704)	–
7	Bua hed	Västra Götaland/ Bohuslän (O)	2015 (1977)	50 (30)	–
8	Kungsbackafjorden	**Halland (N)**	2005	4 740 (499)	FL
9	Utterås	Halland (N)	2010	280 (72)	FL
10/11	Vendelsöarna	Halland (N)	2002 (2015)	1 320 (262)	FL

12	Kullaberg/Västra/Östra	**Skåne (M)**	1986 (2012)	200+160=360 (980)	FL
13	Domsten–Viken	Skåne (M)	2011 (1984)	30 (15)	–
14	Falsterbohalvöns havsområde/ Måkläppen	Skåne (M)	1992 (2011)	41 250 (1 569)	–
15	Hallands Väderö	Skåne (M)	1998	1 510 (339)	FL
16	Knähaken (Municipal MNR)	Skåne (M)	2001	1 360 (0)	–
17	Södra Lommabukten	Skåne (M)	2008	200 (54)	MB Ban on fisheries
18	Foteviken	Skåne (M)	2011	1 940 (730)	FL
19	Bunkeflo strandäng (Municipal MNR)	Skåne (M)	2010	490 (1546)	–
20	Södra Bjärökusten	Skåne (M)	2013 (2003)	4 720 (280)	–
21	Grollegrund (Municipal MNR)	Skåne (M)	2012	1570 (0)	–
22	Jonstorp-Vegeåns mynning	Skåne (M)	2014	1 280 (237)	–
23	Tromtö	**Blekinge (K)**	2011	920 (374)	–
24	Eriksberg	Blekinge (K)	2008	90 (780)<br>20 fresh water	–
25	Gö	Blekinge (K)	2008	1 440 (500)	–
26	Utklippan	Blekinge (K)	2011	1 700 (13)	–
27	Tjärö	Blekinge (K)	2012 (1976)	220 (83)	–
28	Stärnö–Boön	Blekinge (K)	2014	705 (195)	–
29	Värnanäs	**Kalmar/ Småland (H)**	1997	1 450 (102)	MB Ban on fisheries
30	Norra Tjusts skärgård	Kalmar/ Småland (H)	2012	2 170 (241)	MB
31	Beijershamn	Kalmar (H)	2009	220 (162)	–
32	Södra Malmön	Kalmar (H)	2007	1 280 (398)	–
33	Rågö	Kalmar (H)	2013 (1978)	630 (217)	MB
34	Runnö	Kalmar (H)	2008	1 850 (532)	–

35	Kvädöfjärden	**Östergötland (E)**	2007	3 260 (1 267)	FL
36	Bråviken	Östergötland (E)	2011	8 820 (425)	–
37	Kopparholmarna	Östergötland (E)	2012	300 (28)	–
38	Torrö	Östergötland (E)	2008	880 (200)	MB/FL
39	Missjö	Östergötland (E)	2014	1 660 (174)	–
40	Svensksundsviken	Östergötland (E)	2015 (1981)	1 300	–
41	Askö	**Södermanland (D)**	2007	5 220 (624)	MB/FL
42	Salvorev–Kopparstenarna (Underwater reserve)	**Gotland (I)**	1987	56 030 (0)	–
43	Ålö–Rånö	**Stockholm (AB)**	2002	1 760 (1 063)	FL
44	Fifång	Stockholm (AB)	2005	1 480 (237)	FL
45	Nåttarö	Stockholm (AB)	2008	5 950 (609)	FL
46	Villinge Boskapsö	Stockholm (AB)	2012 (1987)	230 (106)	–
47	Stora Nassa	Stockholm (AB)	2013 (1965)	2 660 (280)	–
48	Arholma-Idö	Stockholm (AB)	2015	1 330 (538)	–
49	Gräsö östra skärgård	**Uppsala (C)**	2012	53 400 (1 344)	FL
50	Skaten–Rångsen	Uppsala (C)	1998	1 860 (363)	–
51	Gåsholma	**Gävleborg (X)**	2007	310 (89)	–
52	Salen	**Västernorrland (Y)**	2008	520 (185)	MB Ban on
53	Långören	Västernorrland (Y)	2015	390 (11)	MB
54	Holmöarna	**Västerbotten (AC)**	1980	20 580 (2 800)	FL
55	Kronören	Västerbotten (AC)	2002	4 470 (1337)	–
56	Örefjärden–Snöanskärgården	Västerbotten (AC)	2012	46 810 (740)	FL

**Total**				**330 735 ha, if MNP included: 368 695 ha**	

Number of marine and terrestrial nature reserves (MNRs and TNRs) by coastal county in Sweden, 2015*

Table S2 The acronyms of the coastal counties are compiled, and their geographical positions are marked in Figs. 2a and 2b of the main paper. The total number of TNRs in coastal county is 2 865, whereas the country total of TNRs are 4 284

**Coastal county**	**BD**	**AC**	**Y**	**X**	**C**	**AB**	**D**	**I**	**E**	**H**	**K**	**M**	**N**	**O**	**Country total**
**MNR**	0	4	2	1	2	6	1	1	6	6	6	12	3	7	56
**TNR**	355	274	161	163	141	216	134	124	211	181	96	234	162	413	2 865 (4 284)

***Marine and terrestrial protected areas are according to IUCN divided in different management categories** (Dudley 2008)**:**

I Strict protection [Ia) Strict nature reserve and Ib) Wilderness area];

II Ecosystem conservation and protection (i.e., National park);

III Conservation of natural features (i.e., Natural monument);

IV Conservation through active management (i.e., Habitat/species management area);

V Landscape/seascape conservation and recreation (i.e., Protected landscape/seascape); and

VI Sustainable use of natural resources (i.e., Managed resource protected area).

Marine and Terrestrial Protected Areas in the neighboring countries of Sweden

The large difference in number between reported marine and terrestrial protected areas (MPAs and TPAs of all categories) applies to most countries, including the neighboring countries of Sweden.

Table S3 In 2008, according to the World Bank Indicator–Biodiversity and Protected Areas, the number of MPAs and TPAs are shown below. Source: UNEP-WCMC as compiled by the World Resources Institute, based on data from national authorities, national legislation and international agreements

**Country**	**MPAs**	**TPAs**
Denmark	52	3 847
Finland	15	6 046
Germany	21	14 388
Norway	17	1 795
Sweden	477	4 622

Fact Box

MPAs related to HELCOM, OSPAR and the EU

The regional marine commissions, for example, HELCOM for the Baltic Sea and OSPAR for the Northeast Atlantic have established a joint network of MPAs. This network has objectives and criteria for implementation in line with the EU Natura 2000 network, and should be ecologically coherent (the HELCOM Bremen Declaration 2003). HELCOM MPAs (formerly Baltic Sea Protected Areas, BSPAs) and OSPAR MPAs are designated MPAs and lack protection, unless legally protected according to the national law of the Contracting Parties. In 2014, Sweden had designated 28 HELCOM MPAs (out of a total of 174) and 10 OSPAR MPAs (out of a total of 77). Some of these have been legally protected as marine nature reserves and are all inside the territorial boundary. The HELCOM MPAs and OSPAR MPAs designated by Sweden in the Economic Exclusive Zone (EEZ) are Natura 2000 sites. Similar regional MPA networks have been established by the United Nations Environment Programme/Regional Seas Programme (UNEP-WCMC 2008), and also in Australia (Fernandes et al. 2005) and in the USA (Gleason et al. 2010).

The EU Natura 2000 network protects a large number of core breeding and resting sites for rare and threatened species, and some rare natural habitat types, many of which are protected in their own right, according to the EU Birds and Habitats directives. Natura 2000 sites are either Special Protection Areas according to the Birds Directive or Sites of Community Interest according to the Habitat Directive. In 2011, most areas are designated as SACs (Special Areas of Conservation) with conservation plans. Natura 2000 is another kind of MPA, which has a strong protection according to the Environmental Code of Sweden, but they are not fully protected. The Natura 2000 sites are only fully protected if they are located in a National Park or MNR, and most marine Natura 2000 sites are entirely or partly located in a MNR. In 2014, Sweden had 489 marine Natura 2000 sites according to the Environmental Code (seven of them in the EEZ) and 3 976 terrestrial Natura 2000 sites. Many HELCOM and OSPAR MPAs include Natura 2000 areas, but also other aspects of marine nature conservation, including special objectives and criteria (HELCOM 2013; 2015; OSPAR 2015).

MPAs and TPAs by CBD region

In 2010, out of 190 nations and territories, only 12 with marine jurisdictions had MPA coverage of 10% or more, which is the Convention of Biological Diversity (CBD) target for 2020 (Torepova et al. 2010). Also, the MPA coverage is rather patchy (Fig. S1), with some areas that are already far ahead of the 2020 target of 10% ocean protection, whereas others are far behind.

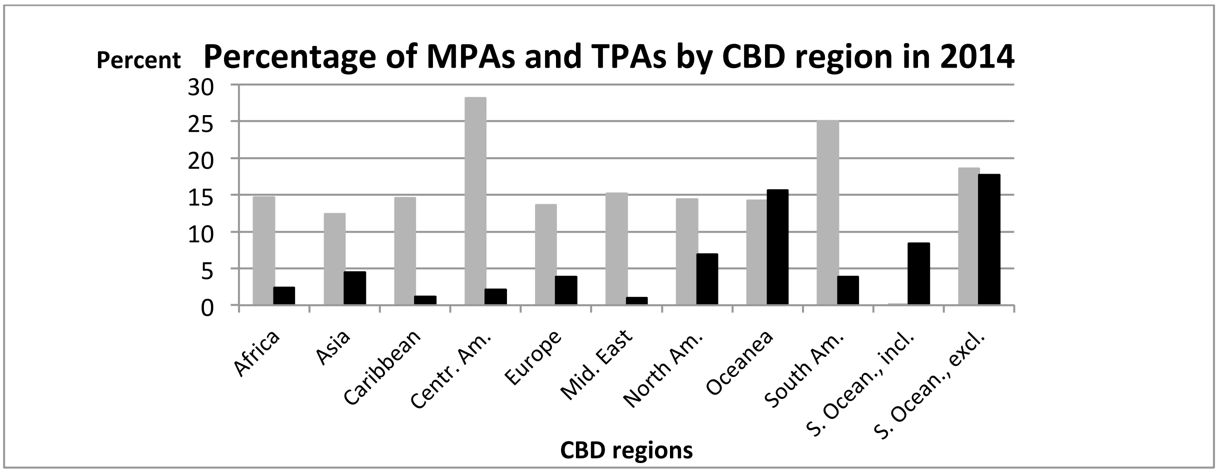

Figure S1 Histogram showing percentage of land covered by protected areas in the CBD-regions (gray) and percentage of marine area (0–200 nautical miles) covered by protected areas in the regions (black). (S. Ocean. incl. Ant. = South Ocean, including Antarctica; S. Ocean., excl. Ant. = South Ocean, excluding Antarctica). Source Deguignet et al. (2014).

In October 2016 the Commission for the Conservation of Marine Living Resources (CCAMLR) reached consensus on a New Zealand/United States proposal to establish a large-scale marine protected area in the Ross Sea region of Antarctica (Hallet 2016). The Agreement will enter into force on 1 December 2017. The Ross Sea region MPA covers 1.55 million km^2, of which 1.12 million km^2, or 72%, is fully protected (no fishing is permitted). It is the world's largest MPA

Appendix S2

Prerequisite of jurisdiction and legislation – the UN Law of the Sea (UNCLOS)

According to UNCLOS, sometimes referred to as the Constitution of the Sea, each coastal state has sovereignty over its territorial sea (12 Nm from the baseline) (Fig. S2). The outer control area of the coastal state is basically its Exclusive Economic Zone (EEZ), and its Continental Shelf. Outside the EEZ follows The Area (the seabed) and the High Seas (the water column above the seabed). The rules of the seabed are not always the same as applies to the water column above. The coastal state does not have sovereignty over its EEZ and Continental Shelf, but it has important rights and responsibilities. The right to control is very strong, especially with regard to the use of resources, environmental protection, including marine wildlife conservation, and marine scientific research (Schiffman 2008). The resources of the EEZ belong to the coastal state. If other countries want to extract the resources in the EEZ, or explore the area, they need permission by the coastal state.

A strong reason for the low number and late start of establishments of MPAs is apparently that the needed international and national legal marine framework on rights and responsibilities of coastal states was not in place until after 1994, when the 1982 United Nations Convention on Law of the Sea (UNCLOS) entered into force (Kelleher and Kenchington 1992). In Sweden, the administrative boundaries, and thereby the responsibilities in the sea of the coastal municipalities and the CABs, was not in place until the 1990s (DSH 1989). After 1994, it became legally clear how the coastal states could manage the use of their territorial seas, EEZ and continental shelves (Jacobsson 2009). After 1994, there is an increased rate of establishment of MNRs in Sweden and MPAs globally (Figs. 3a and 5a in the main paper). Hence, a prerequisite to implement MPAs is legislation for proper enforcement.

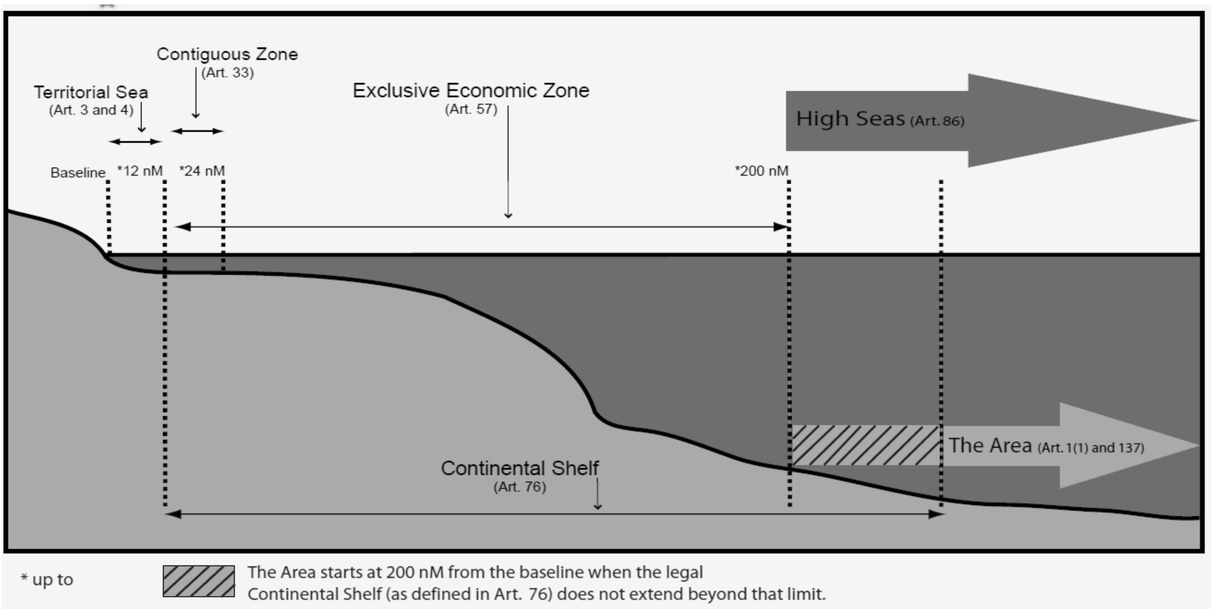

Figure S2 Graphical representation that shows the boundaries and zones of the Sea, in accordance with UNCLOS. Source: UNCLOS.

The Sea is jurisdictionally divided into Inner Waters (inside the baseline), the Territorial Sea (12 nautical miles (nM) from the baseline), a Contiguous zone (a possible additional zone 24 nM from the baseline, claimed by some countries), the Exclusive Economic Zone, EEZ, (200 nM from the baseline) and the High Seas (beyond the EEZ). The Area includes the seabed and ocean floor and subsoil thereof, beyond the limits of national jurisdiction. It starts at the 200 nM or at the end of the Continental Shelf, where this extends beyond the 200 nM boundary (DSH 1983:1). Maritime boundaries delimiting various maritime zones, for example, in semi-enclosed seas, such as the Baltic, are subject to special rules under UNCLOS

High Seas Marine Protected Areas

The use of MPAs in the high seas that lie beyond the jurisdiction of any nation, our common open-access resources have yet to be incorporated formally into international law (Baker et al. 2001). The World Conservation Congress in Hawaii 2016 supported the 2015 United Nations General Assembly Resolution 69/292, on the Development of an international legally binding instrument under the UNCLOS, on the conservation and sustainable use of marine biological diversity of areas beyond national jurisdiction. The resolution covers marine genetic resources, including questions on the sharing of benefits, measures such as area-based management tools, including marine protected areas, environmental impact assessments and capacity-building and the transfer of marine technology.

The 1992 Rio Conference – The UN Conference on Environment and Development
The Law of the Sea left numerous unanswered questions. The convention set no detailed environmental standards for protecting the marine environment. The 1992 United Nations Conference on Environment and Development (UNCED) in Rio de Janeiro added a new theoretical overlay to the 1982 UNCLOS and attempted to further develop international thinking and commitments relating to ocean and coastal decision-making (VanderZwaag 1996). Agenda 21, the 40 chapter action plan for achieving sustainable development, contains Chapter 17 focused on the oceans. Chapter 17 is devoted to sustainable development, including conservation of marine living resources and marine environmental protection in all kinds of seas. Also, at UNCED 1992, the CBD and the Convention of Climate Change was concluded.

Much as a response to the 1992 UNCED, with a number of principles of relevance for ocean and coastal area management, the new 1992 Helsinki and OSPAR Conventions, relevant to Sweden, and the United Nations Environment Programme/Regional Seas Programme began to address issues of biodiversity and marine protected areas. Since then, the European commissions have together with the EU and its Birds and Habitats directives, also adopted in 1992 (92/43/EEG), but with some delay on marine application. It jointly stimulated and put pressure on their Contracting Parties and member countries to act on marine nature conservation. Furthermore, the use of principles and instruments for marine governance and management, for example, marine spatial planning and ecosystem-based management emerged.

In 1995 in Jakarta, at its second Conference of the Parties (COP/CBD) the ministerial statement involved the Jakarta Mandate on conservation and sustainable use of marine and coastal biological diversity. The multi-year work programme of the mandate, including the establishment of marine and coastal protected areas, was adopted by COP/CBD in 1998. These meetings and other related conferences and meetings in the 1990s and 2000s contributed to raise the importance of marine conservation and sustainable use of marine and coastal biological diversity (Nordic Council of Ministers 1995; 1996; SEPA 1997). Apparently, this has stimulated the efforts to designate and establish MNRs in Sweden and MPAs globally, from the 1990s up to present, as reflected both in number (Figs. 3a and 5a in the main paper) and extension of MNRs and MPAs (Figs. 3b and 5b in the main paper).

In our study, we have found that these new commitments have raised the importance of marine nature conservation and influenced the willingness to designate and protect sensitive marine environments (Appendix S1, Fact Box), for example:

- The Swedish commitments to the CBD with the 1995 Jakarta mandate (CBD 2000) on the global consensus on the importance of coastal and marine biological diversity;
- The new 1992 Helsinki and OSPAR conventions with new provisions for nature conservation and biodiversity; and
- The Swedish membership of the EU in 1995 with the Birds and Habitats and other Directives and policies, as well.

As a support and follow up on the Chapter 17 of Agenda 21 and the following UNCED and CBD meetings, in the 1990s and 2000s, the UN Agencies dealing with oceans and coastal issues took several initiatives aimed at improving coordination, coherence and effectiveness related to the oceans. For example, the Sub-committee on Oceans and Coastal Areas of the Administrative Committee on Coordination (ACC SOCA) was formed in 1993. In 2003, an Oceans and Coastal Areas Network, named *UN-Oceans* was established (United Nations 2003). The UN-Oceans Task Force on "Marine Protected Areas and Other Area-based Tools" was established in 2007, in order to strengthen collaboration and coordination among UN organizations dealing with marine protected areas. In particular, this applied to achieve the World Summit on Sustainable development 2012 MPA targeting on representative networks of marine protected areas.

References S1 and S2

Baker, M., B. Bett, D. Billet and A. Rogers. 2001. The status of natural resources on the high-seas – an environmental perspective. WWF/ IUCN, Gland Switzerland.

CBD 2000. Convention on Biological Diversity: The Jakarta Mandate – from global consensus to global action. The CBD Secretariat, Montreal, Canada.

Deguignet, M., D. Juffe-Bignoli, J. Harrison, B. MacSharry, N. Burgess and N. Kingston. 2014. United Nations list of protected areas. UNEP–WCMC, Cambridge, United Kingdom.

Dudley, N. (ed.) 2008. Guidelines for applying protected areas management categories. IUCN, Gland, Switzerland.

DSH 1983. Beslut om havet. [Decisions on the Sea]. The Swedish Marine Resources Commission, DSH 1983:1. Göteborg, Sweden (in Swedish).

DSH 1989. Svensk havsresursverksamhet på 1990-talet. [Swedish Marine Resources activities in the 1990s]. The Swedish Marine Resources Commission, DSH 1989:2. Göteborg, Sweden (in Swedish).

Fernandes, I., J. Day, A. Lewis, S. Slegers, B. Kerrigan, D. Breen, D. Cameron, B. Jago et al. 2005. Establishing no-take areas in the Great Barrier Reef: Large-scale implementation of theory on marine protected areas. *Conservation Biology* 19: 1733–1744.

Gleason, M., S. McCreary, M. Miller-Henson, J. Ugoretz, E. Fox, M. Merrifield, W. McClintock, P. Serpa and K. Hoffman. 2010. Science-based and stakeholder-driven marine protected area network planning: A successful case study from north central California. *Ocean and Coastal Management* 53: 52–68.

Hallett, C. 2016. Nations agree to create world´s largest marine reserve in Antarctica. Science 354: 530.

HELCOM 2013. HELCOM Protect – Overview of the status of the network of Baltic Sea marine protected areas. Helsinki Commission, Helsinki, Finland.

HELCOM 2015. HELCOM MPA database. HELCOM marine protected areas website. Helsinki Commission, Helsinki, Finland.

Jacobsson, M. 2009. Folkrätten, havet och den enskilda människan [International Law, the Sea and the individual man]. Liber, Stockholm, Sweden (in Swedish).

Kelleher, G. and R. Kenchington. 1992. Guidelines for Establishing Marine Protected Areas. A Marine Conservation and Development Report. IUCN, Gland, Switzerland.

Nordic Council of Ministers 1995. Marina reservat i Norden, Del I [Marine reserves in the Nordic countries, Part I]. Nordic Council of Ministers, TemaNord 1995: 553. Copenhagen, Denmark (in Swedish).

Nordic Council of Ministers 1996. Marina reservat i Norden, Del II [Marine reserves in the Nordic countries, Part II]. Nordic Council of Ministers, TemaNord 1996: 546. Copenhagen, Denmark (in Swedish).

OSPAR 2015. 2014 Status report on the OSPAR network of marine protected areas. OSPAR Commission, London, United Kingdom.

Schiffman, H. S. 2008. International law and protection of the marine environment. New York University School of continuing and professional studies, New York, USA.

SEPA 1997. Marina reservat [Marine nature reserves]. Swedish Environmental Protection Agency, Report 4693. Stockholm, Sweden (in Swedish).

Toropova, C., R. Kenchington, M. Vierros and I. Meliane. 2010. Benefits and challenges of MPA strategies. In *Global ocean protection: Present status and future possibilities,* 11–24, ed. C. Torepova, I. Meliane, D. Laffoley, E. Matthews and M. Spalding. IUCN WCPA, Gland, Switzerland.

UNEP–WCMC 2008. National and Regional Networks of Marine Protected Areas: A Review of Progress. UNEP-WCMC, Cambridge, United Kingdom.

United Nations 2003. UN Ocean. Terms of Reference (resolution 68/70, Annex). United Nations, New York, USA.

VanderZwaag, D. 1996. Sustainable development in the maritime sector. Ocean law and policy challenges. Dalhousie Law School and Associate, Oceans Institute of Canada. Halifax, Canada. (Paper presented at a symposium by the Organization of Eastern Caribbean States in St. Lucia 1996).
